# Catchment effects of a future Nordic bioeconomy: From land use to water resources

**DOI:** 10.1007/s13280-020-01391-z

**Published:** 2020-09-14

**Authors:** Eva Skarbøvik, Philip Jordan, Ahti Lepistö, Brian Kronvang, Marc I. Stutter, Jan E. Vermaat

**Affiliations:** 1 Norwegian Institute of Bioeconomy Research, P.O. Box 115, 1431 Ås, Norway; 2 School of Geography and Environmental Sciences, Ulster University, Coleraine, UK; 3 Finnish Environment Institute (SYKE), Latokartanonkaari 11, 00790 Helsinki, Finland; 4 Department of Bioscience, Aarhus University, Vejlsøvej 25, 8600 Silkeborg, Denmark; 5 Environmental and Biochemical Sciences Dept, James Hutton Institute, Aberdeen, UK; 6 Faculty of Environmental Sciences and Natural Resource Management, Norwegian University of Life Sciences (NMBU-MINA), Ås, Norway

**Keywords:** Bioeconomy, Ecosystem services, Long-time monitoring data, Mitigation measures, Sustainability, Water quality

## Abstract

In the future, the world is expected to rely increasingly on renewable biomass resources for food, fodder, fibre and fuel. The sustainability of this transition to bioeconomy for our water systems depends to a large extent on how we manage our land resources. Changes in land use together with climate change will affect water quantity and quality, which again will have implications for the ecosystem services provided by water resources. These are the main topics of this *Ambio* special issue on “Environmental effects of a green bio-economy”. This paper offers a summary of the eleven papers included in this issue and, at the same time, outlines an approach to quantify and mitigate the impacts of bioeconomy on water resources and their ecosystem services, with indications of useful tools and knowledge needs.

## Introduction

As the world is moving towards the end of the era of fossil fuel, a sustainable bioeconomy is envisioned to be our common future solution where food, fodder, fibre and fuel will increasingly be provided by renewable resources (European Commission 2012, 2018). As expressed by the Nordic Council of Ministers (2017): “the bioeconomy is all-encompassing and comprises those parts of the economy that make responsible use of renewable biological resources from the land and water for the mutual benefit of business, society and nature”. However, since the concept is under development, consensus about what “bioeconomy” in fact entails is limited (Golembiewski et al. 2015; Bugge et al. 2016; O’Brien et al. 2017). This makes the consequences of the bioeconomy on the environment and society even more difficult to predict. Policy makers have so far paid little attention to the sustainability of the possible implementation of the concept (Bugge et al. 2016), but several scientists have elucidated the likely environmental impacts of this so-called ‘green shift’ (Ollikainen 2014; Pfau et al. 2014; Eyvindson et al. 2018, Stegmann et al. 2020).

In a review of 87 papers on bioeconomy and sustainability, Pfau et al. (2014) found that the problem most often mentioned was competition for land caused by an increased demand for biomass resources. The amount of land needed for a future bioeconomy remains undetermined since bioeconomy monitoring systems are not yet developed (O’Brien et al. 2017), and the society as well as its technology are under continuous development (Ollikainen 2014; Nyström et al. 2019). However, based on 15 science studies made for the European Commission, Harrison (2010) estimated that an area of 4.5 million ha, approximately the size of Denmark, would be needed to fulfil EU’s goal that 7% of the need for liquid fuel should come from biofuel production. As it is not unlikely that the future need will exceed 7%, also fallow, marginal or lower productivity lands may be used for biomass production in order not to compete with agricultural land (Sheppard et al. 2011). This demand for land entails a conflict between bioeconomy and the recent incentives for sustainable intensification of agriculture to feed a growing human population (Tilman et al. 2011; Rockström et al. 2017). Moreover, the increased use of marginal lands may give rise to other conflicts since they are often valuable for biodiversity and other natural functions acting as ecosystem services (Dale et al. 2010).

A less studied challenge is the impacts of land use changes created by bioeconomy purposes on water resources, including water quantity, quality and aquatic ecology. The review by Pfau et al. (2014) reported studies that predicted extreme damage to natural ecosystems, enhanced eutrophication, increases in pests and invasive species, as well as a high demand for water that would affect aquatic ecosystems. Despite this, 6 years ago these authors found only five relevant published papers that mainly addressed bioeconomy-related challenges to water systems. This demonstrates that bioeconomic impact on water resources, including their various ecosystem services, is an understudied field of science.

One of the main water quality goals of the EU Water Framework Directive (WFD; European Commission 2000) is to achieve Good Ecological Status (GES) for all water bodies, and its § 12 prescribes that new activities leading to the degradation of water bodies should either be prohibited or subject to management restrictions. The potential conflicts between the WFD goals and the emerging land-based bioeconomy led to the creation of BIOWATER, a Nordic Centre of Excellence.^1^ The centre’s main objective is to quantify the combined bioeconomy-related effects of land use change, climate change and industrial innovation on carbon, nutrient and water cycles as well as on major ecosystem services, including good ecological status of fresh waters. As the title of this special issue highlights, we aimed to take stock of these possible impacts. Inspired by the high interest experienced when preparing for the 2019 conference on land use and water quality impacts (LUWQ 2019), we decided to compile an up-to-date assessment of the possible effects on water of bioeconomy, with contributions both from within and outside our consortium. In this, we must emphasise that this scientific topic involves a high degree of uncertainty and that BIOWATER has two more years to run. Accordingly, our currently presented results and views will be complemented in future publications.

The main purpose of this summary paper is to outline our approach to assess the impacts on water resources of the emerging bioeconomy, to discuss the elements of such an approach by referring to the papers in this special issue, and to point to future knowledge needs for its implementation. However, given the uncertainties of how bioeconomy may affect land use, waters and human welfare, we first introduce the results of a questionnaire to scientists, managers and students about their visions of bioeconomy, which we organised at the BIOWATER Special Session at the LUWQ conference in 2019.

### How do fellow scientists and managers perceive a future bioeconomy?

The many unanswered questions regarding the future direction of bioeconomy, including the amount of land needed, its use, its sustainability and modelling of different Nordic scenarios, helped to shape a questionnaire for the LUWQ 2019 special session of BIOWATER. The participants coming from different countries and continents were asked to answer the following four questions:

- What would bioeconomy mean in your country or region?
- Which land use changes would have a positive effect on water quality?
- In your country/region, which energy source would dominate in a world with bioeconomy?
- Which type of model can best simulate the future bioeconomy at catchment scale?

**Fig. 1:**
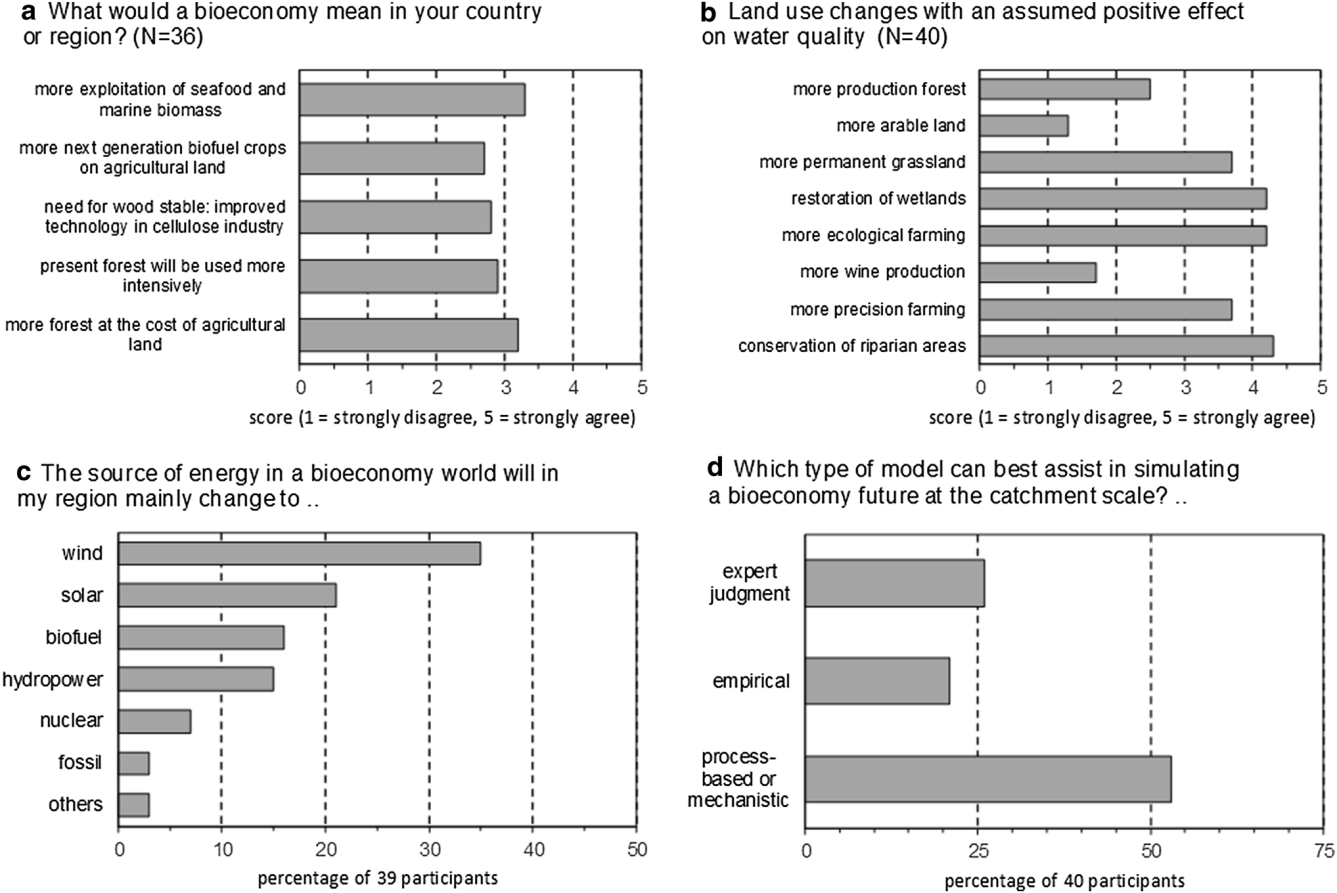
Results of the four questions of the BIOWATER questionnaire at LUWQ 2019

Their answers (Fig. 1) illustrate the current thoughts and ideas of forty scientists, students and water managers.

The participants regarded a bioeconomy-based future to be most strongly connected with increased exploitation of marine biomass and more forest at the expense of agricultural land (Fig. 1a). However, the other three options also had an average score > 2.5, indicating that all five options are likely. This further points to the uncertainty regarding future land use and that any of the five options presented are likely in a world with more bioeconomy.

Most participants voted that conservation of riparian areas would lead to a better water quality, followed by restoration of wetlands and organic farming (Fig. 1b). Precision farming and permanent grassland were also high on the list of land use changes that could lead to improved water quality.

To the question of where our future energy will derive from, most answered wind (35%), followed by solar energy (21%), biofuel (16%) and hydropower (15%) (Fig. 1c). As expected, fossil fuel was not expected to persist in the future, and neither was nuclear energy.

Lastly, scientists are often asked to model the future world as forecasting ‘green shift’-induced land use changes combined with a changing climate is of great importance. To the question of which predictive catchment models would be most well suited for this purpose, mechanistic/process-based model types emerged as the favourite (53%). Moreover, many participants suggested that use of ‘expert judgement’ would be a feasible way of making prognoses for a future with bioeconomy (26%) (Fig. 1d).

## Study area and approach

### The Nordic countries as a case

BIOWATER is exploring the environmental consequences of the transition to bioeconomy using four Nordic countries as a case study area (Sweden, Norway, Finland and Denmark). The Nordic countries co-operate on several arenas, including the political, economic and cultural, and, according to the Nordic Council of Ministers,^2^ this co-operation is the world’s oldest regional partnership. The “Nordic approach” and co-operation generate added value for the countries and people of the region, but it should be noted that marked differences exist between the countries.

**Fig. 2:**
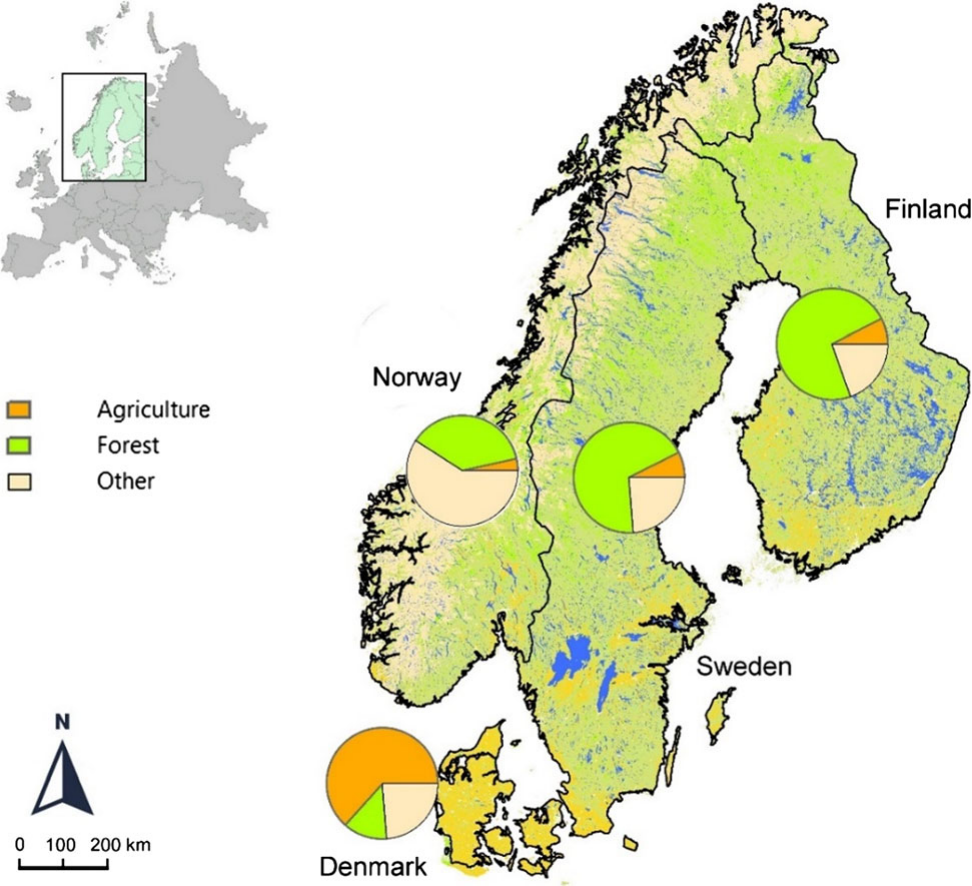
Land use cover in the Nordic countries today. “Other” includes mountains above the treeline, open uncultivated land, peatland, urban areas, freshwaters. Land use cover (%) derived from Marttila et al. (2020)

This is not least true when it comes to topography, soils and, hence, land use (Fig. 2), which again implies that each country’s conditions for establishing bioeconomy are different. Finland has the largest proportion of forest land use (70%). About one-third of Finnish forests are found in peatlands, whereas Swedish (ca. 69%) and Norwegian (ca. 37%) forests are more often located on shallow mineral soils. In Denmark, forest land use covers only approximately 13%; instead Denmark has the highest agricultural land use, more than 60% of the land being cultivated. In contrast, the agricultural land use of Finland and Sweden is 7–8% and that of Norway is only 3%. This also means that the potential for land use changes differs between the countries as, for example, shallow moraine over bedrock (large parts of Norway) cannot readily be transformed to agricultural land. This obviously has consequences for the relative importance of, for example, forest versus agriculture for the provision of bio-economical resources.

This highly variable landscape platform that we use for making assessments about a future bioeconomy in the Nordic countries complicates our analyses, but the variability simultaneously ensures that our research results will be relevant for numerous other regions and countries of the world.

### Methodological approach

**Fig. 3:**
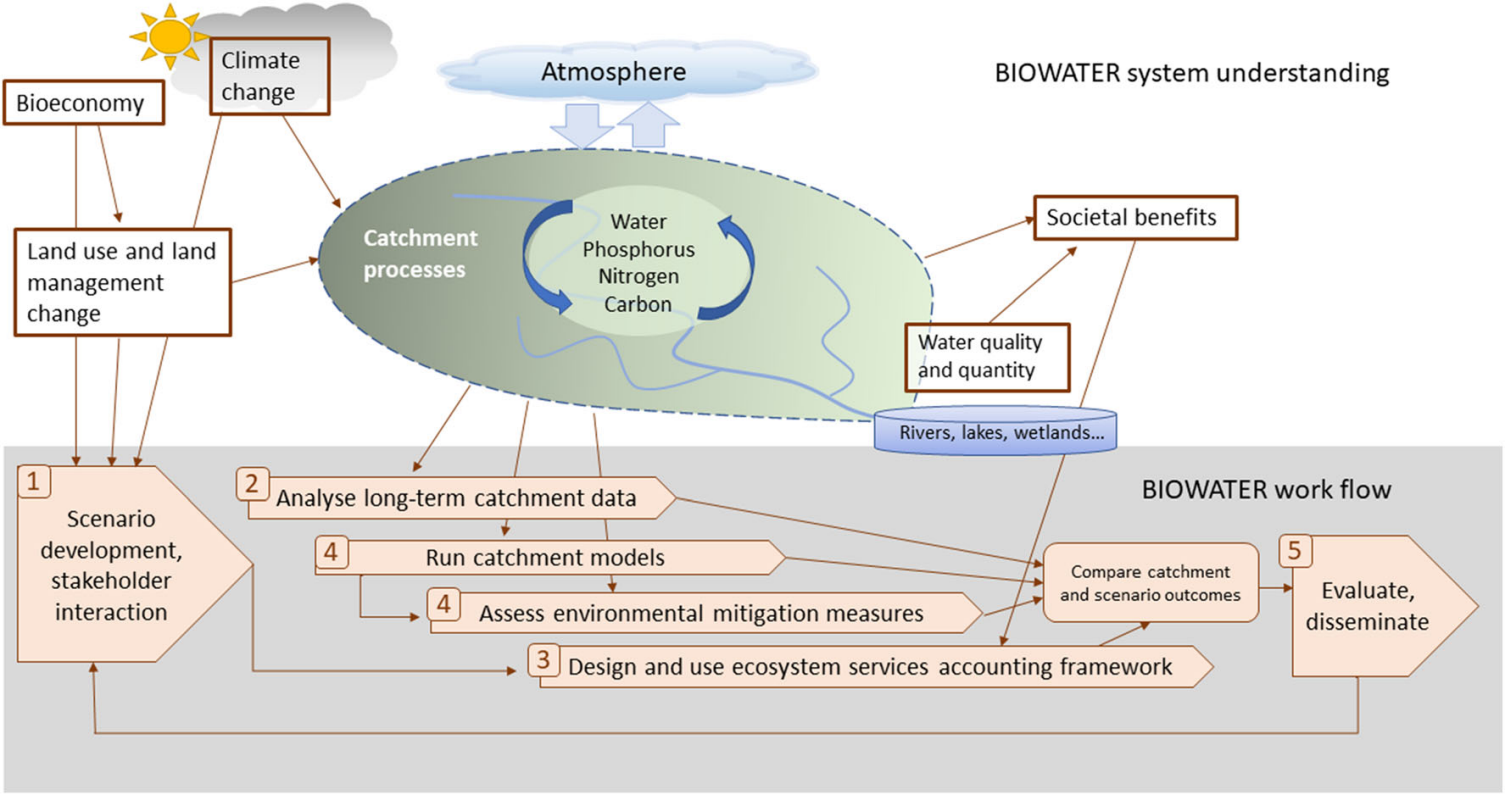
The structure of BIOWATER functions as a methodology for assessing impacts of the bioeconomy on water resources. The numbers refer to module numbers and are reflected in the elements listed in Table 1 and the headings of the discussion of the respective elements

BIOWATER’s envisaged structure (Fig. 3) combines the elements of a comprehensive methodology to explore the possible effects of the bioeconomy on water resources. The numbers on the thick arrows at the bottom of the figure represent the centre’s scientific modules. These modules constitute elements of our framework approach that flows from the design of scenarios, to system understanding at several, parallel levels and to evaluation and dissemination. Certainly, the use of these elements is in itself not novel, but their interdependence and combined adjustment to catchments with changing land use make the approach ‘fit for purpose’. Furthermore, by working with these elements, we can pin-point the knowledge gaps that need to be filled for a better prediction of the impacts of the green shift for water resources and their ecosystem services.

The elements are repeated in Table 1 together with the relevant paper(s) in this special issue that discuss the specific elements. In the following, each element will be discussed in more depth based on the papers of this special issue and other relevant literature.

**Table 1:** Overview of elements in the BIOWATER approach to assess impacts on bioeconomy on water resources and their ecosystem services. The element numbers refer to the modules of BIOWATER, as shown in Fig. 3. BIOWATER results from element/module 5 have not yet been published. Note that some of the papers cover several elements

Step	Elements of the approach	Explanation	Representation in papers in this SI^a^
1	Scenario development, stakeholder interaction	Projecting plausible futures of possible land use changes under the bioeconomy. Involving stakeholders in the articulation of plausible options for land use	Rakovic et al.
2	Analyse long-term catchment data	Understanding the system by utilising long-term datasets on water quantity and quality in combination with records of land use and land management variations	Kaste et al. Marttila et al. Skarbøvik et al. Sundnes et al. Wenng et al.
3	Design and use ecosystem services accounting framework	Assess impacts on society of bioeconomy by identifying the supporting, provisioning, regulating and cultural services of water resources	Vermaat et al.
4	Run catchment models^b^	Modelling effects on water resources by using catchment models to predict water quality under a variety of different land use, land management and climate conditions	Djodjic et al. Hashemi and Kronvang Vermaat et al.
4	Assess environmental mitigation measures	Mitigating the impacts by developing cost-effective mitigation measures and land use planning tools to reduce losses of nutrients, pesticides and soil	Blankenberg and Skarbøvik Carstensen et al. Djodjic et al. Hashemi and Kronvang
5	Evaluate, disseminate	Elaborating a synthesis of the above steps, ensuring stakeholder interaction, disseminating information to managers and policy makers	–

^a^*Ambio* Special issue. 2020; 49(11)

^b^Complete catchment models, where a full set of alternative land use options have been modelled to assess water quantity and quality, are not yet finalised in BIOWATER; the papers listed do, however, make use of catchment models to solve specific issues

## Scenario development for a Nordic bioeconomy [element #1]

Societies need to prepare for expected changes in climate, economic systems, land use and management, and quantitative abstractions of possible future alternatives are of political interest. This is the subject area of scenarios, a field that has grown and matured hand-in-hand with climate science. As the future is inherently unknown, one should work out plausible and consistent projections of alternative scenarios. We do not have such projections yet for how the Nordic bioeconomy might develop. Rakovic et al. (2020) have therefore set out to articulate an existing set of five benchmark societal scenarios, based on O’Neill et al.’s (2017) Shared Socio-economic Pathways (SSPs), into narratives for the Nordic context, termed Nordic Bioeconomy Pathways (NBPs). Each NBP follows the SSP’s core meaning so that SSP 1, which is delineating a sustainable future, is also the most sustainable scenario of NBP 1, and so on.

The work on scenarios is ongoing in BIOWATER, with articulation of land use options together with stakeholders, and translation of qualitative scenarios to numerical storylines that can be used as inputs in catchment models. Although the most comprehensive modelling of catchments has not yet been finalised, a first partial use of the NBPs has been done by Vermaat et al. (2020), and they are also considered in Hashemi and Kronvang (2020) and Djodjic et al. (2020).

## Analyse long-term catchment data [element #2]

Catchment hydrological and biogeochemical understanding has developed using empirical monitoring and experimental manipulation since the 1960s (e.g. the Hubbard Brook Ecosystem Study^3^; Likens et al. 1978). Many long-term catchment and river monitoring networks exist in Nordic countries today (e.g. de Wit et al. 2007; Bechmann and Deelstra 2013; Skarbøvik et al. 2014; Stålnacke et al. 2014; Tattari et al. 2017; Räike et al. 2019; Hashemi et al. 2020). Biophysical data support the analyses of change over time for many land cover types and their pressures. Coupling this with land management information over increasing time periods supports linkages between land use and water quality. However, data collection networks have generally been designed with a historical problem-solving focus, such as monitoring eutrophication or acid rain effects. They may therefore not always be suitable to answer questions regarding new challenges across regions and timescales, an example being the effects of expansion of bioresource productive land uses, such as forestry, on water environments. Several papers in this special issue explore the utility of current long-term datasets in this new topical context. In particular, the ability of the data to explore relationships between land use change and climate change is addressed. This is a critical time for the future bioeconomy where (1) the need for food security contrasts uncomfortably with (2) the potential for water quality (and other environmental) impacts, both potentially being shaped by the influences of a changing climate.

Climate change can affect the hydrometeorological drivers of diffuse pollution from catchments, which strongly interacts with land use change and together shape water impacts. This is recognised throughout the world for hydrology and water resources (Hagemann et al. 2013; Donnelly et al. 2017), for land use change (climate as an agent of change; and land use change as a climate mitigation strategy) (Nelson et al. 2014; Searchinger et al. 2018) and for diffuse pollution patterns and projections (Ockenden et al. 2017; Mellander et al. 2018). The contribution by Marttila et al. (2020) sets the scene of this theme with recognition of the land use—water quality—climate change nexus and a call for improved understanding of catchment-scale water and elemental fluxes. Learnings from long-term data series are essential to fully understand the long-term consequences of policy decisions that are currently in play or deemed as urgent.

Another relevant aspect is the need for standards to compare with: for example, what nutrient loads are acceptable against natural background values? Across most of Europe, reference conditions are set for most water types under the umbrella of the WFD, and environmental goals are often determined based on these. Exceedance of these environmental goals implies that water authorities must implement often expensive measures (Hering et al. 2010). However, as demonstrated by Skarbøvik et al. (2020), these reference conditions are not well established for all Nordic lowland streams, and the uncertainty implies a need to revisit this important instrument for water quality management based on a combination of appropriate spatially consistent data and modelling. Sundnes et al. (2020) point out that the measures to capture carbon through forestry programmes have received much attention in Nordic countries, gaining political commitment since 2015. The authors review the knock-on water quality consequences to this intensification and find good practice to avoid impacts in the short term. What is further required, however, is a longer-term trade-off analysis between carbon sequestration caused by forest land use intensification and subsequent water quality impacts that are assessed across regions.

In seven small agricultural catchments, Wenng et al. (2020) investigated water quality impacts of different land uses by analysing data over a 30-year period. Relationships between longer growing season and reductions in river *N* concentrations were found in catchments used for cereal production, but not in grassland catchments, which points to a complex series of environmental responses that are still only partially understood. Added to this mix, Kaste et al. (2020) point out that reductions in river nitrogen concentrations can be linked to reductions in atmospheric nitrogen depositions over a similar period. Collectively the studies show the advantages of deeper exploration within, and between, monitoring data records for ‘added value’ insight into relationships between parameter groups and other informative (e.g. non-linear trend) behaviours; this is one aspect in Wenng et al. (2020).

This indeterminacy within the land use—water quality—climate change nexus means that extrapolations between regions and scales for modelling purposes will remain a challenge. Deciphering all these links as the bioeconomy evolves and the climate changes over the next years and decades will require an empirical underpinning. Going forward, while longer-term data series as currently captured in Nordic countries were designed for specific purposes at the time of initiation, the analysis provided by the contributions highlights an opportunity for reflection on future priorities. Marttila et al. (2020) finishes by identifying knowledge gaps and the need for improving models with sound empirical understanding—that can be provided with longer-term time-series data. The opportunity is to adjust or re-design programmes that account for new challenges across the Nordic countries based on the findings from BIOWATER, ideally while maintaining the data record consistency and utility for existing and continuing issues. Harmonising this across themes such as land–water–air connections (e.g. Kaste et al. 2020; Wenng et al. 2020), improvement of data certainty (e.g. Skarbøvik et al. 2020; Vermaat et al. 2020) and informing the development of environmental impacts and mitigation measures (e.g. Carstensen et al. 2020; Djodjic et al. 2020; Sundnes et al. 2020) is a requirement for bioeconomy data gathering in a changing climate.

## Design and use ecosystem services accounting framework [element #3]

Biogeochemical catchment data and models may well fall short in the effects on the many different and often competing ways society is benefitting from its natural environment. Computational models that also attempt to encompass sociological, economic and possibly other societal dimensions end up being highly abstract or generic (e.g. an early example in Zuchetto 1975) and their outcomes can be hard to trace and difficult to translate back into policy measures. IPCC, for example, has decided to uncouple scenarios of societal change from those of geophysical global change (van Vuuren and Carter 2014).

Ecosystem services framing can be one approach to integrate various contrasting forms of societal benefits from a landscape—or catchment. Being inherently cross-disciplinary, the concept is still subject to considerable debate, and different analytical frameworks have been designed to address issues of environmental advocacy, comprehensive cost–benefit assessments of policy or land use allocation alternatives, or enhanced revenue extraction (Nelson et al. 2009; Bateman et al. 2013; Bouma and van Beukering 2015). Vermaat et al. (2020) conclude that one such framework can feasibly be used to carry out a scenario comparison. So, this is a tool that we ‘have’ and can use for integration across sectors, an issue that is quite relevant when we compare the possible effects of a developing bioeconomy, where some sectors may lose, and others win.

In the definition from Boyd and Banzhaf (2007, ‘final ecosystem services are components of nature, directly enjoyed, consumed, or used to yield human well-being’), the words ‘final’ and ‘directly’ must be contemplated as an important perspective (cf. Bateman et al. 2011), just as the notion that different services may accrue to very different beneficiaries. Vermaat et al. (2020) adjusted and tested a method from Mononen et al. (2016) combining the most recent benchmark classification (Common International Classification of Ecosystem Services; CICES5.1) with a harmonised land cover classification such as CORINE and locally available statistical data. They termed it the ‘Mononen cascade’ and included it as a simple spreadsheet model that allows for quantification of all relevant ecosystem services provided in a landscape in both biophysical and monetary terms. Several services that can be considered as ‘intermediate’ rather than ‘final’ have been quantified through their effect on other, more distinctly final, services. Examples are pest regulation via crops and stream water temperature regulation via recreative angling.

The effect of changes in land cover due to the Nordic Bioeconomy Pathways (NBPs) from element # 1 (scenario development) was tested for two catchments, the Lillebæk Stream in Denmark and the Halden River in Norway (Vermaat et al. 2020). Land management options were not included at this stage and, consequently, only the large changes in land use were analysed. In the agriculture-dominated Lillebæk catchment, the NBP predicting a more sustainable world with more forest cover and a more diverse agriculture led to a higher estimated total summed benefit (from about 300 to 400 € ha^−1^ [catchment] year^−1^) due to more varied provisioning services and a higher importance of regulating services. In the forest-dominated Halden catchment, only regulating services became more important with an increase in non-drained wetlands, but the summed total benefit did not alter much. The remaining scenarios, including the NBP that is least environmentally concerned, did not lead to large changes in land cover, and effects on ecosystem provision were therefore limited. If the bioeconomy implies increased and more intensive forestry, and the Halden catchment is representative for larger areas of Central Scandinavia, then the effect on ecosystem service delivery is limited. If we instead have a more sustainable development, then a limited increase in regulating services (flood regulation, carbon sequestration, nutrient retention) appears to be realised at the expense of some forest productivity. For the intensively used Danish agricultural catchment of Lillebæk, the effects are quite different and diversification in the sustainable scenario appears to enhance total societal benefit substantially.

The short answer to the question ‘what the effect of the bioeconomy on ecosystem service provision by Nordic catchments would be’ is thus ‘it depends’, and it appears to depend notably on the prevailing land use.

## Run catchment models and assess environmental mitigation impacts [element #4]

Empirical monitoring data and modelling outcomes could give an indication of the future situation and provide knowledge on feasible mitigation measures and adaptation strategies (Giri and Qiu 2016). Catchment simulation models have become increasingly versatile and powerful and often have become a standard to inform management and policy (e.g. Arheimer et al. 2005; Futter et al. 2007; Huttunen et al. 2016). Thus, catchment models that combine hydrology and biogeochemistry have become a precious information source (e.g. Wade et al. 2002; Jackson-Blake et al. 2016). In this special issue, modelling has been done, *inter alia*, by Hashemi and Kronvang (2020) and Djodjic et al. (2020), to optimise the effect of mitigation measures.

Djodjic et al. (2020) studied how to optimise the placement of constructed wetlands (CWs) at catchment scale to reduce phosphorus losses to surface waters. Such CWs are considered an important mitigation measure as increased sediment and phosphorus losses to surface waters from biomass production in both agriculture and forestry might be expected in the future. In a mixed forestry-agriculture catchment in Sweden, they found that optimisation of the positioning and size of CWs had a great potential for reducing the land needed. Possible positive side effects could be increased water retention during floods and likely also on biodiversity, as these CWs represent small habitats of more stagnant waters throughout the catchments.

Another, and more radical, measure is to convert arable land to forest or permanent grassland, so-called set-aside land. Hashemi and Kronvang (2020) studied the multi-functional benefits from such a targeted land use change in a Danish agricultural dominated catchment. This resulted in a method to optimise the spatial allocation of land, taking into consideration national goals on surface water quality, groundwater quality, nature conservation, as well as climate plans. The work revealed that single-target optimisation towards hot-spot areas should be substituted with a more integrated way of multiple-object-targeting. The authors concluded that their method may be used for assessing possible effects of a bioeconomy and can be used to model effects of the Nordic Bioeconomy Pathways (cf., element # 1; Rakovic et al. 2020).

Carstensen et al. (2020) also discussed the need to maximise the effects of mitigation measures, but they also stressed the importance of minimising undesirable by-products. Thus, the management and design of mitigation measures should not solely focus on nutrient reduction, but also take into consideration potential negative by-products such as Green House Gas (GHG) emissions, phosphate releases or reduced biodiversity. Knowledge about the GHG emissions from the different mitigation measures is crucial, and future research on how to reduce such unwanted emissions is needed. These authors focused on measures related to drainage systems, since a combination of climate change and using more marginal lands for biomass production is expected to increase the need for drainage of both agricultural and forested lands. They therefore reviewed the efficiency of mitigation measures targeting nutrient losses from agricultural drainage systems in the temperate regions of the world. They focused on nitrate and total phosphorus removal efficiency of (i) free water surface constructed wetlands, (ii) denitrifying bioreactors, (iii) controlled drainage, (iv) saturated buffer zones and (v) integrated buffer zones. The load of nitrate was substantially reduced by all five drainage mitigation measures (mean: 26–68%), while the measures mainly acted as sinks of total phosphorus—but occasionally also as sources of phosphorus. The study showed that large variations were reported in the removal efficiencies of the mitigation measures and that factors such as design, runoff characteristics and hydrology influenced the performance. The envisaged increase in temperature might improve the performance of the mitigation measures but more intense precipitation events will challenge their hydraulic capacities and, thereby, their performance, with needs for new dimensions in a changing climate.

The need for measures that not only address single issues was also emphasised by Blankenberg and Skarbøvik (2020), who studied in a more integrated manner the functioning of riparian buffer zones in South-East Norway and their importance in the future bioeconomy. They found that buffer zones intended for grass production in general had fewer positive effects (nutrient retention, bank erosion, biodiversity) than the ones with natural vegetation. They concluded that there is a future need for more integrated studies of buffer zones to investigate how to increase their ability to retain nutrients, prevent bank erosion, enhance biodiversity, facilitate recreation and, at the same time, optimise the production of food and fodder without jeopardising water quality.

A future bioeconomy is expected to imply more intensive use of land areas for biomass production while climate change may increase nutrient and soil losses (Jeppesen et al. 2011) and enhance eutrophication (Deelstra et al. 2011; Jeppesen et al. 2012). Mitigation measures targeted to optimise both the reduction of nutrient losses and the production of biomass, while avoiding negative side effects and enhancing positive ones, are the ideal. Given the large amount of mitigation measures and the complexity of natural processes within a catchment, there is ample room for research in this field of science in the years to come.

## Discussion

In addition to providing an overview of the papers of the special issue on “Environmental effects of a green bioeconomy”, our aim with this summary paper was to outline the different elements in a methodological approach to study the possible effects of the green shift on water resources and their ecosystem services. By doing so, we detected knowledge gaps that need to be filled to follow such an approach. The term ‘biobased economy’ has only existed in the last two decades (Golembiewski et al. 2015), and we cannot yet know how this shift in the world’s economy will affect Nordic catchments. Important questions for the future include, *inter alia*, (1) How much land will be needed to provide the necessary biomass for the bioeconomy? (2) To which extent will the need for biomass change the proportion of forests, agricultural land and more marginal lands (e.g. outlying fields, riparian zones, flood-prone areas)? (3) How much intensification will we see in agriculture and forestry? (4) How will these changes then interfere with biodiversity conservation policy objectives?

The large uncertainty of how land use will change further increases the uncertainty of possible adverse environmental impacts on hydrology, water quality and biodiversity (e.g. Pfau et al. 2014; Eyvindson et al. 2018). Hence, we need to be prepared for contrasting perspectives and outcomes, which makes the chosen scenario approach (O’Neill et al. 2017; Kok et al. 2019; Mitter et al. 2019; Rakovic et al. 2020) useful, but not necessarily a one-off exercise. Scenario development will likely become an ongoing exercise as we gradually increase our knowledge about what land use changes we may expect.

Long-term data series are like gold mines for both scientists and managers, as they provide the possibility to detect and learn from interannual trends caused by changes in, for instance, land use and climate, thereby improving our understanding of important landscape processes. As noted by Marttila et al. (2020), future monitoring efforts should also seek to include new monitoring methods such as online sensors (Rode et al. 2016) and more sophisticated modelling tools as also suggested from the participants at the LUWQ special session (Fig. 1d). This would provide more information about the governing factors of catchment processes and can allow for more accurate prediction of future scenarios. However, it is important that this progress in technical solutions for monitoring does not result in a disruption of long time series based on more traditional sampling and laboratory techniques; rather, the two approaches should be maintained in parallel.

While long time series of water quantity and quality exist in both smaller and larger catchments in the Nordic countries, our work has revealed that systematic, long-term data on environmental effects of forestry operations in research catchments are quite limited (Marttila et al. 2020; Sundnes et al. 2020; de Wit et al. unpubl.). On the other hand, there are experimental (paired) catchment manipulations and monitoring of single disturbances that give empirical knowledge about increased export of carbon, nitrogen, phosphorus and suspended solids to water courses following some 10 years after forestry operations (Kreutzweiser et al. 2008). Many such studies report temporary nutrient or carbon exports after logging (e.g. Ahtiainen and Huttunen 1999; Joensuu et al. 2001; Futter et al. 2010; Oni et al. 2014). Recent studies in Finland indicate that considerably longer-term nutrient leaching, of decades, may occur from drainage in peatland forestry to watercourses (Nieminen et al. 2018; Finer et al. 2020). In a Biowater long-term dataset of 69 Nordic headwater catchments, concentrations and fluxes of total nitrogen and total phosphorus were highest in agricultural catchments, intermediate in forestry-impacted and lowest in natural catchments; and forestry-impacted catchments exported on average over 40% more nitrogen than natural catchments (de Wit et al. unpubl).

Long-term datasets, together with models and expert judgement, have been used to determine the reference conditions of Nordic water bodies. The reference conditions are useful benchmarks when the rural landscape changes, but especially in lowland catchments where pristine conditions are difficult to find, the uncertainties are large (Skarbøvik et al. 2020). This also means that environmental goals have uncertainties. Environmental goals determine the amount of mitigation measures needed, and as we move towards a future with both changed climate and land use, it is likely that a new generation of mitigation measures must be developed. This means that there is a need to improve the targeting, precision, cost-effect and cost-benefits of the measures, while at the same time enhancing multiple functions and reducing negative side effects (Blankenberg and Skarbøvik 2020; Carstensen et al. 2020; Djodjic et al. 2020; Hashemi and Kronvang 2020). Increased conflicts between mitigation measures and production of biomass are not unlikely in the future bioeconomy and this calls for studies that minimise the land needed for mitigation measures without compromising the ecological needs.

Mitigating the impacts of forestry is less investigated, but several studies reviewed by Kreutzweiser et al. (2008) demonstrated that stem-only or partial-harvest logging reduced the impacts on nutrient release and exports in comparison to whole-tree clear-cutting. This is less likely in the bioeconomy with strategies to increase biomass production, and the effect of this on water quality at landscape scale is not adequately understood (Laudon et al. 2011). As noted by Sundnes et al. (2020), the long-term effects of forest fertilisation and intensified forestry remain unclear, which is highly unfortunate as we stand on the brink of a future with assumedly intensified use of forest produce. According to Marttila et al. (2020), more knowledge about the impacts of a forest-based bioeconomy on waters is therefore strongly needed, including longer-term datasets and recent empirical evidence on the catchment- and regional-scale impacts.

Land use changes due to the bioeconomy may affect ecosystem services provided by water, as outlined in Vermaat et al. (2020). Their analysis did not include the added effect of climate change impacts, where a combination of warmer, wetter, wilder weather may affect services such as production of clean drinking water, irrigation of crops, flood control and recreation. Whenever water is involved in a service, this may have profound effects, particularly beyond the 2050-time horizon. Other papers in this special issue (e.g. Djodjic et al. 2020; Hashemi and Kronvang 2020) have assessed multiple benefits deriving from a catchment-scale mitigation measure or change in land use, but none have tried to integrate all possible societal uses. Given the simplicity of the ‘Mononen-cascade’ presented in Vermaat et al. (2020), it appears possible to deploy it in these and similar cases.

## Conclusion

This *Ambio* Special Issue is a current stocktaking of possible adverse environmental effects on water systems of a developing Nordic bioeconomy. We outline how each of the 11 papers in this issue fits into the scientific steps of the project approach taken in the Nordic Centre of Excellence ‘BIOWATER’ to better predict bioeconomy effects on water quality and quantity as well as related ecosystem services. We observed that

(i) comprehensive empirical data on water quality, quantity and land use practices are available from long-term Nordic observation series; however, catchments representing different forestry activities are highly underrepresented;
(ii) modelling of possible future effects of bioeconomy requires development of Nordic Bioeconomy Pathways (NBPs) that are included in this issue, but also more specific scenarios for the different agricultural and forestry attributes are required and currently under further development within BIOWATER;
(iii) the applied ecosystem services framework appears to have sufficient resolution to identify changes caused by bioeconomy and trade-offs among different services; and
(iv) better targeting of mitigation measures (location and dimension) offers clear optimisation opportunities for improving surface water quality and can assist in reducing negative side effects of a growing bioeconomy, including unnecessary occupation of fertile land areas useful for production of food, fodder or other biomass products.

While this special issue highlights promising learnings, important knowledge still needs to be gained to improve our understanding of future bioeconomy effects on water resources.

## Footnotes

1 The centre consists of eight Nordic partners in four countries (Denmark, Finland, Norway and Sweden), has collaborating European research institutions acting as advisors, and is financed by NordForsk under the Nordic Programme of Bioeconomy (www.biowater.info).

2 https://www.norden.org/en/information/official-nordic-co-operation.

3 https://hubbardbrook.org/
